# Insights About Circadian Clock and Molecular Pathogenesis in Gliomas

**DOI:** 10.3389/fonc.2020.00199

**Published:** 2020-02-28

**Authors:** Kholoud Arafa, Marwan Emara

**Affiliations:** Center for Aging and Associated Diseases, Zewail City of Science and Technology, Cairo, Egypt

**Keywords:** circadian clock, neuro-glial communication, glioma, cancer hallmarks, chronotherapy

## Abstract

The circadian clock is an endogenous time-keeping system that has been discovered across kingdoms of life. It controls and coordinates metabolism, physiology, and behavior to adapt to variations within the day and the seasonal environmental cycles driven by earth rotation. In mammals, although circadian rhythm is controlled by a set of core clock genes that are present in both in suprachiasmatic nucleus (SCN) of the hypothalamus and peripheral tissues, the generation and control of the circadian rhythm at the cellular, tissue, and organism levels occurs in a hierarchal fashion. The SCN is central pacemaker comprising the principal circadian clock that synchronizes peripheral circadian clocks to their appropriate phase. Different epidemiological studies have shown that disruption of normal circadian rhythm is implicated in increasing the risk of developing cancers. In addition, deregulated expression of clock genes has been demonstrated in various types of cancer. These findings indicate a close association between circadian clock and cancer development and progression. Here, we review different evidences of this association in relation to molecular pathogenesis in gliomas.

## Introduction

**Figure 1:**
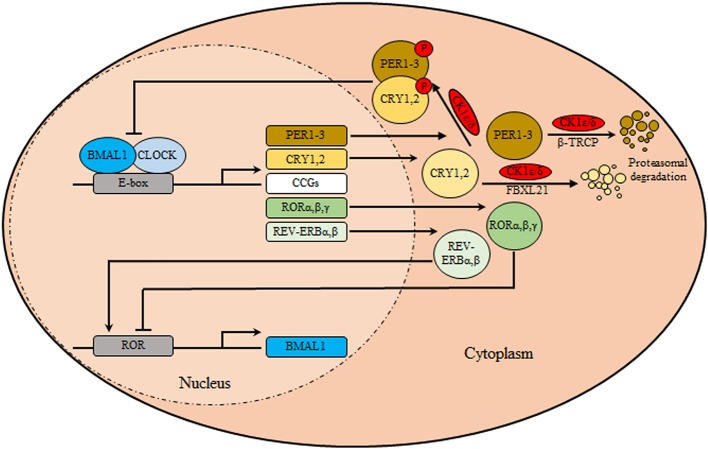
The circadian clock machinery. The core clock machinery consists of two main loops. The transcriptional activators BMAL1 and CLOCK bind to E-box motifs in their target genes promoting the expression of the repressors period (PER1-3) and cryptochromes (CRY1, 2). Upon accumulation, PER/CRY heterodimers are phosphorylated by casein kinase 1δ/ε (CK1δ/ε), and translocate to the nucleus where they inhibit the BMAL1/CLOCK transcriptional activity thus inhibiting their own transcription. Over time, in the cytoplasm, phosphorylation and proteasomal degradation of CRYs and PERs are regulated by CK1δ/ε- F-Box and Leucine Rich Repeat Protein 21 (FBXL21) and CK1δ/ε-Beta-transducin repeats-containing proteins (β-TRCP), respectively, relieving their auto-inhibition, restarting the cycle. In a secondary loop, BMAL1/CLOCK stimulates the transcription of nuclear receptors genes encoding REV-ERBα/β and RORα/β/γ. These are respectively transcriptional repressors and activators that regulate rhythmic BMAL1 expression.

The circadian clock system orchestrates different physio-biochemical and behavioral aspects with the suprachiasmatic nuclei (SCN) as the main coordinator synchronizing all peripheral clocks in the body of mammalians (1). Anatomically, The SCN is a bilateral structure based in the hypothalamus, above the optic chiasm, on both side of the third ventricle. In murine, ~10^4^ neurons are contained in each unilateral SCN and manifested in two anatomic substructures: a ventral (core) area, which borders the optic chiasm, receiving retinal input through the retinohypothalamic tract, and a dorsal (shell)area which surrounds and gets input from the core (2). Besides the neuronal circuitry, the SCN is also composed of glial cells, which contribute to overall pacemaker function. Interestingly, glial astrocytes were found to exhibit circadian expression of clock genes and immunostaining for glial fibrillary acidic protein revealed circadian variations in glial morphology in the SCN (2, 3). Notably, SCN neurons are known to exhibit sustained expression of circadian gene, electrical activity, as well as secretion of neuropeptide independent of the external cues (4). Functionally, the SCN neuronal subpopulations differ in expression of their neurotransmitters, represented by vasopressin (by dorsomedial shell region) and vasoactive intestinal polypeptide (VIP) (by ventrolateral core region) and the inhibitory neurotransmitters gamma aminobutyric acid (by entire SCN population). Moreover, Glutamate is considered the main excitatory neurotransmitters released from retinohypothalamic tract after exposure to photic stimulus. It binds to their cognate ionotropic glutamatergic receptors located in the SCN, namely the N-methyl D-aspartate receptors (NMDA), the activation of which stimulates subsequent changes in clock gene expression (5). At the cellular level, the molecular machinery of the circadian clock (Figure 1) is made of transcriptional translational feedback loops (TTFL) regulated by a set of core/canonical clock factors, namely BMAL1, CLOCK, the cryptochromes (CRY 1, 2), and the period (PER 1-3) constituting intricate positive and negative limbs (6). The positive limb involves BMAL1/CLOCK heterodimers or alternatively BMAL1/Neuronal PAS domain protein 2 (NPAS2) binding to E-boxes cis-regulatory enhancer sequences located in the promoter region of the circadian output genes including *PER* and *CRY* genes (7). In-turn CRY and PER proteins form oligomers, phosphorylated by casein kinase 1δ/ε (CK1δ/ε), transported from the cytoplasm into the nucleus, and suppress their own transcription via inhibiting BMAL1/CLOCK forming the core loop (negative limb) (7). This cyclic process is known to control the period of circadian oscillations. TTFL is further modulated by the activity of CK1δ/ε (8). In the cytoplasm, phosphorylation and proteasomal degradation of CRYs and PERs are regulated by CK1δ/ε- F-Box And Leucine Rich Repeat Protein 21 (FBXL21) and CK1δ/ε-Beta-transducin repeats-containing proteins (β-TRCP), respectively (9). Furthermore, the positive and negative limbs are interwoven as BMAL1/CLOCK also initiates the expression of nuclear receptors genes *REV-ERB*α, β *and ROR*α, β, γ which subsequently can recognize a retinoic acid receptor-related orphan receptor element in the promoter of *BMAL1* thus reducing or enhancing its transcription, respectively, forming the secondary stabilizing loop (7, 9).

Core clock genes and clock-controlled genes have roles pertinent to aging (10), immunity (11), metabolism (12), DNA repair (13), and controlling the cell cycle progression (14). Therefore, aberrant circadian rhythms can eventually lead to sleep disorders, metabolic and inflammatory diseases, and cancer (9, 15). The disruption in rhythmicity can be caused either due to heritable genetic mutation disturbing the normal sleep-wake cycles (e.g., familial advanced sleep-phase syndrome) or environmental external stimulators and life style (e.g., shift workers) (16). Such aberrations of circadian rhythms have been long reported to cause carcinogenesis (17–19). Generally, there are four main aspects through which circadian disruption might lead to carcinogenesis. (1) Circadian clock is an immense regulator of rhythmic gene expression implicated in vast cellular processes including protein folding, metabolism, autophagy, DNA damage repair, and redox regulation. (2) a large number of circadian clock proteins were found to physically interact with oncogenic proteins e.g., c-Myc. (3) Clock proteins and cofactors probe changes in redox state, post-translational processes brought about by oncogenic programs, e.g., hypoxia inducible factor-1α activation, which affect their stability, localization, or function. (4) There is a reciprocal regulation between the circadian clock and several endocrine factors (e.g., cytokines and neurotransmitters) that can be hijacked by cancers leading to circadian disruptions reviewed in Sulli et al. (9), Sulli et al. (16), and Chen-Goodspeed and Lee (20).

Whether the disruption in circadian rhythm is cause or a consequence in tumorigenesis is still debatable. It is conceivable that gliomagenesis would lead to reciprocity in anomalies pertinent to circadian time-keeping and entrainment. Herein, we will focus on the close association between circadian clock and molecular pathogenesis in gliomas through reviewing the circadian clock in relation to different molecular and cellular changes implicated or resulted in glioma pathogenesis.

## Glia and Gliomas

In the mammalian nervous system, glia represent more than half of cells. Together, glia and the central nervous system (CNS) neurons originate from neuroepithelial progenitor cells in the embryonic neural tube and forebrain, where the radial glia, descendants of neuroepithelial progenitor cells transforms into both neurons and microglia (21, 22). Following the generation of neurons and through gliogenic switch, radial glia differentiate into astrocytes or oligodendrocyte precursors (22). In addition, ependymal cells were found to originate from radial glia (23). Unlike other glia in the CNS, microglia is a transformed primitive macrophages that is generated from hematopoietic stem cells, that has migrated to the CNS (24). Collectively, the glial cells of CNS are presented by astrocytes, oligodendrocytes, microglia, and ependymal cells. Functionally, astrocytes serve as the main connective tissue in the brain. Alongside, oligodendrocytes mainly perform the specific function of axonal myelination in the CNS. Whereas, microglial cells exercise restricted immune function and may have functions in tissue restoration and repair (25). Molecular circadian clock has been reported in ependymal cells (26), microglia (27, 28), and astrocytes (4, 29, 30). No evidence supports the presence of internal circadian clock in oligodendrocytes (31, 32), however, clock genes could regulate the proliferation of oligodendrocytes precursor cells in the hippocampus (33).

The vast variation in cellular linages in glial cells leads to heterogeneity of the associated malignancies known as gliomas which constitutes over 70% of all brain malignancies and represent the deadliest brain tumors (34, 35). Recently, the World health Organization (WHO) has reclassified the gliomas subtypes (Pilocytic astrocytoma, diffuse astrocytoma, oligodendrogliomas, anaplastic astrocytoma, anaplastic oligodendrogliomas, glioblastoma, and ependymoma) based on a defined set of genetic mutations (35).

## Neuro-Glial Communication in Fine-Tuning the Circadian Rhythm

The proof of SCN neuronal circuitry controlling glial rhythmicity has been evidently demonstrated using cortical astrocytes obtained from *mPeriod2::luciferase (Per2::luc)* knock-in mice and *mPeriod1::luciferase (Per1::luc)* transgenic rats indicated that the PER-based oscillation in astrocytes weakens when the neuronal signals are lacking/absent. Rhythms could be regained after replacing the culture medium or treating the cultured cells with the Calcimycin (calcium ionophore) or Forskolin (adenylate cyclase agonist). A pulse-dependent phase shift was induced following media change and re-initiation of rhythmicity. Sustained rhythmicity (7 days or longer) was observed in a portion (~30%) of astroglial cultures when co-cultured with SCN explants (4, 36). Conclusively, astroglial cultures behave as weakened circadian oscillators that rely on signals of neurons to maintain oscillations of individual cell or to synchronize glial cell clock populations (36). However, a paradigm shifting study using astrocyte-specific genetic complementation in the SCN of mice lacking both neuronal CRY genes showed that SCN astrocytes can autonomously encode circadian information and relay the initiation and sustainment of such patterns to their neuronal partners suffering an incompetent TTFL clock. Where glutamate gliotransmitter controls circuit-level circadian time-keeping in CRY1/2-null SCN expressing glial fibrillary acidic protein-restricted CRY1. Nevertheless, temporally the effects on Per2::luc oscillations of astrocyte-restricted CRY1 took appreciably longer (7 days) to initiate rhythms relative to neuronal counterparts (2 days) (37).

**Figure 2:**
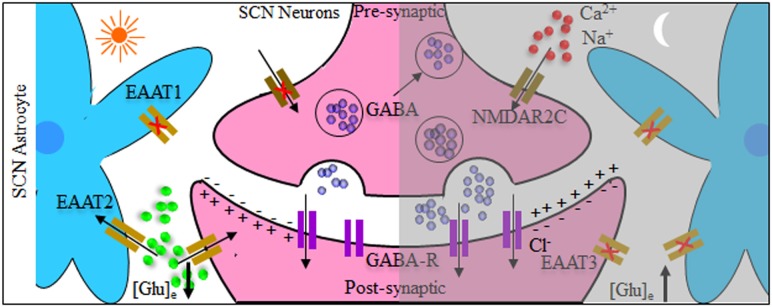
Tripartite model system of the suprachiasmatic nucleus (SCN) neurons and astrocytes. Both neurons and astrocytes have different temporal activity in circadian day and night. During circadian daytime, clearance of extracellular glutamate ([Glu]e) takes place by reduced astrocytic release and increased intracellular uptake by glutamate transporters [excitatory amino acid transporters 1–3 (EAAT1–EAAT3)]. This reduces the GABAergic tone across the network, leading to depolarization and increased electrical neuronal firing within the suprachiasmatic nucleus (SCN). The reverse happens during circadian night where [Glu]e levels increase, caused by astrocytic release as well as reduced activity of glutamate EAAT transporters. Subsequently this activates pre-synaptic NR2C subunit-containing NMDA-type glutamate receptors (NMDAR2C), increasing the pre-synaptic intracellular Ca^2+^ concentration ([Ca^2+^]i) facilitating inhibitory neurotransmitter GABAergic release, and suppressing postsynaptic neurons electrical activity.

In an attempt to mechanistically explain the neuro-glial synchronous contribution to the final rhythmic fluctuations, it has been shown that SCN circuit-level timekeeping results from an interdependent astrocytic-neuronal signaling (38). This was fortified by the data depicting that a remodeling in the circadian behavioral rhythms in adult mice was induced by somatic genetic re-programming of intracellular clocks in SCN astrocytes. Such effect was attributed to glutamatergic gliotransmission from SCN astrocytes which were manipulated *via* inhibition of neuronal glutamate transporters, excitatory amino acid transporter 3 leading to a disrupted Per2::luc bioluminescence. Mechanistically, astrocytic activity during circadian nighttime reciprocally suppressed the activity of SCN neurons through regulating the levels of extracellular glutamate ([Glu]e) which was detected by neurons of the dorsal SCN specific pre-synaptic NMDA containing NR2C subunits (NMDAR2C) (39, 40), where NR2C is a subtype of NR2-NMDA receptor (41). Astrocytic intracellular calcium ([Ca^2+^]i) oscillations had a key role in this model which is reminiscent of the results mentioned earlier in the drosophila model. It is worth mentioning that the astrocytes in Drosophila and brain of mammals are extremely similar in regard to their morphology and molecular signatures suggestive of functional conservation. This intricate cycle of timely regulation of neuronal activity is depicted in Figure 2. Additionally, an astrocyte-targeting strategy (*via* tamoxifen induction at the glutamate-aspartate transporter promoter) to induce that circadian disruption in a sub-population of glutamate-aspartate transporter-positive astrocytes. The perturbation of such population yielded a global effect on the neuronal clock in the brain, where the removal of *BMAL1s* from murine astrocytes led to a BMAL1 global reduction in the SCN through gamma aminobutyric acid signaling (42).

The prominence of neuro-glial co-ordination in pacing the circadian behavior is done through employing different molecules. In drosophila model, EBONY, an enzyme functioning to conjugate β-alanine to several neuroactive modulators, played the significant role in such communication. It is expressed in a discrete subpopulation of glia where it exhibits circadian oscillation in expression. The disruption of EBONY glial circadian expression led to arrhythmic locomotor activity even though the circadian cycling of the upstream circadian genes *PER* and *Timeless* (*TIM*) were normal indicating a normal neuronal output (43, 44). Furthermore, another study emphasized the effect of calcium dependent gliotransmission in the cross-talking with neurons and contribution to time keeping. Utilizing the same model organism, conditional modulation of glial-specific vesicle trafficking, membrane ionic gradient, and calcium signaling imposed arrhythmic locomotor activity. Also, upon perturbing glial cells conditionally, reversible effects were observed on pigment-dispersing factor, an important clock neuropeptide transmitter revealing the capacity of glia-to-neuron signaling in controlling the circadian circuitry (30). Of note, the pigment-dispersing factor secreting neurons, anatomically allocated at large ventral-lateral (LNvs), are crucial for clock entrainment and resynchronization. Both glial and neuronal and clocks are recruited to configure the architecture of the LNv projections along the day, thus maintaining a precise structural and functional plasticity of the circadian network. Remarkably, the glial clock disruption affected LNv structural plasticity irrespective of the functional output (45). Taken together, such studies underscore the important contribution of the glial system in regulating circadian behavioral rhythms. Along the same line, glial contribution to the mammalian circadian behavior has been proven. Intriguingly, astrocytes in the SCN deemed responsive to light stimulation via increasing the expression of the early immediate *FOS* gene (46), emphasizing their potential involvement in the entrainment of circadian machinery to photic zeitgebers. Additionally, VIP induces astrocytic clock gene induction, ATP release (29). Such modulation might globally affect circadian machinery. Deploying mice model, astrocytes daily rhythms within the SCN determine the period of wheel-running activity. In a breakthrough study, through targeted ablation of *BMAL1 clock* gene in SCN astrocytes using CRISPR-Cas9 technology led to lengthening the circadian period of clock gene expression in the SCN and in locomotor behavior. Comparable results were obtained from SCN astrocytes with short-period CK1ε *tau* mutation (47). This may demonstrate that astrocytes within the SCN population communicate to neurons to maintain robust circadian rhythms. A detailed insight on the contribution of different glial cells on the regulation of the circadian system is reviewed (39, 48).

## The Clock Genes Expression and Glioma

Deregulated circadian clock genes are involved in gliomagenesis. The expression levels of the core circadian *clock* gene in the high-grade glioma was significantly enhanced relative to the low-grade glioma and non-gliomas (49). Data showed that there are differential patterns of expression in the clock genes in glioma cells relative to their paired neighboring normal brain tissues signifying asynchrony amongst the circadian clocks. The relevant finding was confirmed by another study, in which the chromosomal number alteration was identified using SNP-array. Results specified the amplification at 4q12 chromosomal region where the mammalian *CLOCK* gene is located. The copy number alteration on the DNA level affected the gene mRNA levels as well which suggests a strong correlation to the pathogenesis of the disease (50). Similarly, clinic-based and case-control study has shown overexpression of glioma *BMAL1* in comparison to normal brain (51).

Furthermore, abnormalities in expression of *PER1* and *PER2* are associated with the occurrence of glioma. PER1 and PER2 oscillation profiles were found to vary between both glioma and normal brain tissue, albeit being within the circadian range in both. Where the mRNA and protein levels of PER1 were significantly reduced in higher grade gliomas relative to low grade ones (52). The findings lend support to the tumor suppressive action of *PER* genes that needs to be tuned down in cancerous cells. Also, through using luciferase reporter genes the core clock gene PER2 exhibited a circadian transcriptional activity in C6 glioma cell lines. Moreover, it was found that profiles of the temporal expression as well as the phase relationship of the different mRNA rhythms of clock genes including *PER3, CRY1, BMAL1, REV-ERB*α, and *DBP* were conforming to those in the SCN. However, *PER1/PER3* expression in C6 glioma cells deemed unresponsive to Ca^2+^- influx stimulation, meaning that the C6 circadian oscillation got detached from neighboring neuronal signaling (53). From the signaling perspective, the treatment of C6 cells with noradrenaline stimulated a temporary increase in *PER1* mRNA expression as well as protein level via activation of β2 -adrenergic receptors. Specifically, noradrenaline induction of *PER1*mRNA expression relied on Src/Glycogen synthase kinase 3β pathway among other cascades of less significant contribution (54). Similar to *PER* genes, lower expression of *CRY1* and/or *CRY2* was observed in gliomas compared with their matched healthy tissues. Particularly, the intensity of immunostaining for CRY2 between high-grade gliomas and low-grade gliomas was significantly lower (55). Similar results were reported for the circadian *TIM* gene (56).

Among the core circadian genes with antitumor activity is the transcription factor NPAS2a paralog for CLOCK forming heterodimers with BMAL1 and contributes to the circadian oscillations. Using genome-wide mapping, KDELR1 was identified as a direct transcriptional target of NPAS2. KDELR1 is known to be responsible for the retrieval of the endoplasmic reticulum chaperones as well as intracellular signal transduction and has been reported to be deleted in glioma (57, 58). Such finding further confirms the intricate relation between circadian system and gliomagenesis. However, based on individual-level information obtained from The Cancer Genome Atlas, it was found that the overexpression of *NPAS2* variants bearing single nucleotide polymorphism (SNP) were associated with glioma-related mortality (51).

All the aforementioned transcriptomic and mechanistic studies confirm that in normal cells the circadian clock exercises strong control over multiple hallmarks of cancer. The disrupted circadian genes expression might be at the nexus of the aberrant processes underpinning the glioma pathogenesis (9).

## The Clock and Glioma Stemness

Cellular proliferation follows a circadian metronome; meaning, proliferation rate varies diurnally. Similarly, stem cells abide by the structured cell cycle phases being either continuously or partially proliferating systems. For the latter group, cells enter into a dormant state (G0 phase), during which circadian heterogeneity plays a critical role in dictating the fate of the cell through controlling differentiation promoting pathways (59). Accordingly, stem cell chronobiology might be instrument when it comes to cancer treatment (60). Taking this into account might help evade the resistance of cancer stem cells (CSCs) against cancer therapeutics. Such difficulties might arise due to multiple factors including CSCs slow cycle, expression of high levels of drug export proteins, and their independence of oncoproteins that stand as main targets of novel chemotherapeutics (61). Aberrant gliogenesis might be the core cause for gliomagenesis. From a developmental biology point of view multipotent neural stem cells give rise to glial stem cells (GSCs) through a process known as neurogenic-gliogenic switch. Subsequently, GSCs differentiate into divergent glial lineages. In this capacity given, the normal brain developmental process gets hijacked, GSCs might serve as the nucleus for gliomagenesis (35). Thus, studying of circadian rhythms status in during neurogenesis and gliogenesisis essential to better understand gliomagenesis. Importantly, it was recently reported that neural stem progenitor cells (NSPCs) obtained from hippocampal area (SVZ and DG) of adult mice were self-sufficient clock cells with innate capacity of producing circadian rhythms. This conclusion was investigated for and validated by immunocytochemistry for mPER1 protein that was found to be localized to the inner, more stem cell-like neurosphere core. Intriguingly, the targeted deletion of *BMAL1*^−/−^ directed cell differentiation to glia rather than neurons (62). This highlights the prominence of circadian genes in dictating the cellular fate of progenitor cells. The C6 line was invaluable for isolating and studying glioma stem cells as well as glioma stem-like cancer cells (GSLCs). It was reported that the monolayer of a subpopulation of C6 cells, a morphologically distinct H33 negative population, was devoid of circadian nuclear localization of mPER2. Unlike C6 monolayer, C6 tumorspheres enriched in GSCs retained functional circadian machinery suggesting that the tumorspheres microenvironment supported circadian oscillations (63, 64).

The glioblastoma heterogeneity might be partially imparted by the phenotypic plasticity indigenous to CSCs promoting versatility required for tumor growth (65). Glioblastoma cells or their associated subpopulation of CSCs are capable to trans-differentiate into endothelial- like cells that can replace the host-derived endothelial cells necessary for forming a tumor-associated vasculature (66). Such findings pave the road for another vital process for cancer survival known as epithelial mesenchymal transition (EMT) discussed hereafter.

## The Clock and Epithelial Mesenchymal Transition (EMT) of Glioma Cells

Circadian rhythms can influence the progression and severity of cancer via controlling the EMT process in tumor cells. It has been illustrated that perturbing the circadian oscillation of EMT regulators within the cancerous niche including Transforming Growth factor β (TGFβ) and Glycogen synthase kinase 3β led to hindering cancer development (67, 68). Studying glioma model, EMT was initiated by growing C6 cells in serum-free stem cell medium (SCM) containing growth factors [epidermal growth factor (EGF), fibroblast growth factor (FGF), Platelet-derived growth factor (PDGF)-alpha-beta]. Following initiation of EMT, which was validated by expression of *ZEB1* and vimentin, the small tumorspheres began to form. As the number of post-EMT cells increased in a tumorsphere circadian oscillation was observed with a period duration range, 19–29 h. Rhythms were present independent from the synchronization signal using forskolin treatment. It was concluded that endogenous circadian oscillators control EMT in gliomas enhancing larger population of post-EMT cells at a particular time of day (69). Neurotransmission at the neural-tumor synapse has been implicated as a causative factor in gliomagenesis through monitoring the impact of serotonin (5-HT) secretion on glioma cells morphology profile throughout 48 h. It was found that the response profile exhibited a circadian rhythm with peak response to precede the nadir of the previously reported daily rhythm in 5-HT levels in rat brain model. Morphologically, the cells resembled post-EMT cells suggestive of enhanced invasiveness. This effect was proposed to be mediated by the 5-HT induced change in the intracellular Ca^2+^ profile (70).

**Figure 3:**
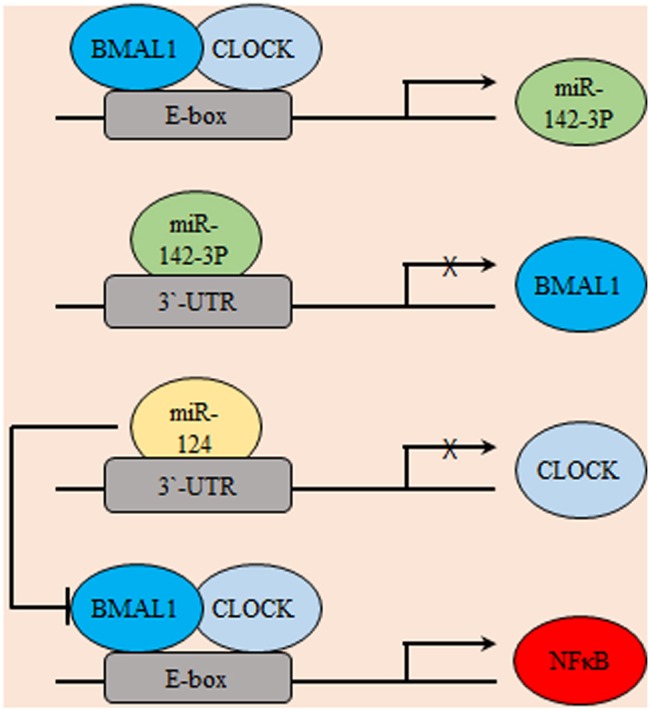
Gliomagenesis suppressive effects of microRNAs. Several micro-RNAs were found to be exhibit circadian expression including miR142-3p. In-turn, miR142-3p inhibits the transcription of its activator BMAL1. Similarly, miR-124 transcriptionally represses CLOCK. Consequently, the potential pro-oncogenic effects of NF-κB are indirectly suppressed.

In coherence with previous reports, the glioma stimulating effect of the core circadian gene, *CLOCK* was emphasized. The observed up-regulation of *CLOCK* might be a consequence of down-regulated miR-124 in glioma which was frequently reported to inhibit tumor cell proliferation and migration through targeting specific genes like Slug, Twist, and Snai2. Results showed that miR-124 can suppress the expression of *CLOCK* by directly targeting its 3′ untranslated regions (3′UTR). Mechanistically, results illustrated that restoration of miR-124 or silencing of CLOCK in glioma cell decreased the nuclear factor kappa B (NF-κB) activity, which suggested a potential miR-124/CLOCK/NF-κB axial relationship in gliomagenesis (71). Similarly, the miR-142-3p and miR-142-5p can be produced by pre-mir-142, where miR-142-3p can target its activator, BMAL1 suggesting a potential negative feedback loop that includes the core clock genes and miRNAs (Figure 3) (72). REV-ERBβ is a variant of REV-ERBα family of transcription factors which has redundant functions in regulating circadian rhythm, metabolism, and inflammatory response. REV-ERBα is more abundant in normal tissues while, REV-ERBβ is the major variant in various human cancer cells (73). REV-ERBβ expression was detected in human glioma specimens of different grades and was found to positively correlate with the malignancy phenotype. *REV-ERB*β expression promoted proliferation, migration, and invasion of glioblastoma multiforme (GBM) through transcriptional upregulation of AXL receptor tyrosine kinase (AXL), an EMT key regulator. AXL/Phosphoinositide 3-kinases (PI3K)/protein kinase B (AKT) axis mediated the tumorigenic effects of REV-ERBβ. In addition, REV-ERBβ knockdown remarkably diminished the maturation of focal adhesion and downregulated proteins participating in actin nucleation and polymerization required for effective cell migration (74).

## The Clock Relation to Angiogenesis and Invasiveness of Glioma

The processes of angiogenesis and invasiveness are inseparable. As compiling studies reported that upon suppressing angiogenesis tumors alternatively switch to invasiveness and metastasis for survival (75). By clawing their way through neighboring tissues, initially hypoxic core cancer cells can easily access preexisting tissue vasculature. Such adaptive behavior is evident by the increased invasion and local metastasis noticed in human GBM under antiangiogenic therapies (76, 77). Circadian system acts as a barrier against pathological tumor vasculature development, the disruption of which poses as one of the most important steps that facilitates tumor angiogenesis. Amongst the key players in promoting tumor metastasis is the hypoxic microenvironment of the cancerous niche. Increasing evidence supports the existence of bidirectional regulation between the clock and the hypoxia-inducible factors (78). One recent study has highlighted the importance of hypoxia-clock signaling in gliomagenesis. This was done through copy number and transcriptomic profiling of 32 circadian clock genes to point outputative loss-of-function and gain-of-function of clock genes in various cancer types including glioma. Results proved that the expression of clock genes with putative tumor suppressive properties (Clock_Loss_) is inversely correlated with tumor hypoxia, the downregulation of this gene set resulted in significantly higher rates of mortality in glioma (*P* < 0.0001). The results suggested that both hypoxia and circadian pathways can be co-regulated. It should be mentioned that both pathways rely on structurally analogous transcription factors containing PER-ARNT-SIM (PAS) domains implying possible interaction (79).

Of relevance is the anti-infiltratory role played by the entrainment regulators pituitary adenylate cyclase-activating polypeptide (PACAP) and VIP under hypoxic condition (80, 81). This effect occurs through indirectly reducing *HIF* and *EGFR* expression, which are considered as key modulators of cell migration and angiogenesis. These peptides act via the inhibition of PI3K/AKT and mitogen activated protein kinase (MAPK)/extracellular signal-regulated kinases (ERK) signaling cascades, controlling hypoxia-inducible factors (82). Mechanistically, these findings were corroborated by another study mentioning that the anti-proliferative activity of PACAP and VIP is mediated via the PACAP type I receptor. However, independent of c-AMP signaling cascade in C6-glioma cells (83). For C6 cells under caloric restriction PACAP and VIP augmented the reduction of CYCLIN-D1 and BCL-2 levels and increased the expression of *P53* and cleaved *Caspase-3* (84). On a side note, PACAP was found to have rhythmic oscillation in the C6 glioma cell-line. Where, a thyroid-specific transcription factor 1, activates the *PACAP* promoter in a dose-dependent manner. In transcriptional hierarchy, thyroid-specific transcription factor 1 promoter activity was found to be inhibited by CLOCK and BMAL1, probably via recruiting different co-repressors (85). Another key player of cellular invasiveness is inositol requiring enzyme-1 (IRE1, with α and β isoforms), one of the endoplasmic reticulum stress sensors activating the mammalian unfolded protein response pathway. In GBM, it was found that the inhibition of IRE1α affects cellular invasiveness capacity via triggering modulations of the extracellular matrix as well as hindering the cellular ability of proliferation, migration, and adherence. Dominant negative construct of *IRE-1*α expression in a GBM cell line (U87) impairs angiogenesis of U87 derived-tumor xenograft and that can be rescued by restoring of the IRE-1α/XBP1 pathway activity. The regulation of *PER1* mRNA by IRE-1α is a key molecular event that is capable of controlling glioma angiogenesis, invasion, and growth. This was suggestive of the possibility that the circadian clock could impact on several cellular processes through its connection to the mammalian unfolded protein response (15).

As inferred from the previous study, that several circadian factors as well as few casein kinases, are components of the endoplasmic reticulum stress system. They are involved in the cell cycle control, cell proliferation processes, and apoptosis. Specifically, the expression levels of casein kinases, which phosphorylate PER proteins and affects its subcellular localization, were found to be affected by the blockade of Endoplasmic Reticulum-Nuclei-1, the key endoplasmic reticulum stress sensor. This pathway was aggravated under glutamine, glucose deprivation, and hypoxia suggesting a direct relation to the tumor metabolic microenvironment (86).

It has been demonstrated that *BMAL1* is down-regulated in particular types of cancer (87) and its knockdown increased cell proliferation and tumor growth in cell culture and mice, respectively (88). An inverse relationship between BMAL1 levels and glioma invasiveness was previously reported. In glioma cells, cell invasion was inhibited by *BMAL1* overexpression through blocking of PI3K/AKT/matrix metalloproteinase-2 (MMP2) pathway where BMAL1 attenuates *BCL-W* oncogene that activates the invasion pathway (89). Accordingly, BMAL1 may act as a tumor suppressor, inhibits the growth and invasion of cancer cells (89).

Another important controller of mammalian circadian machinery is the tumor suppressor p38 MAPK. In C6 glioma cells, p38 MAPK inhibition with SB203580 showed to lengthen the period of the *Per2::luc* reporter. P38 MAPK activation is also under rhythmic control in neural SCN, glial cells, and in peripheral fibroblasts cells. Up-regulated expression and activity of p38 MAPK is a bad prognostic marker in GBM. Unlike normal HA glial cells, phosphorylated p38 MAPK levels were high and arrhythmic in invasive IM3 glioma cells. Despite this arrhythmicity in p38 MAPK phosphorylation, timed application of p38 MAPK activity inhibitor VX-745 to IM3 cells at time-points corresponding to low activity ofP38 MAPK in HA glial cells reduced the invasive properties of IM3 cells (90).

**Figure 4:**
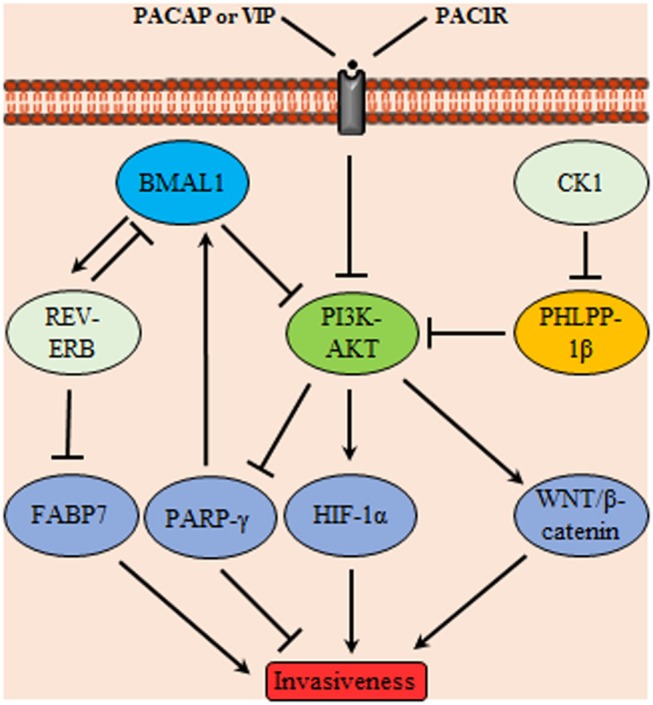
Glioma invasiveness control through a signaling network mediated by circadian proteins. Phosphoinositide 3-kinases (PI3K)-protein kinase B (AKT) controls glioma invasiveness by stimulating HIF-1α and WNT/β-catenin signaling; it also inhibits peroxisome proliferator-activated receptors (PPARγ) Upstream controllers of PI3K-AKT are the circadian proteins BMAL1, PH domain leucine-rich repeat protein phosphatase (PHLPP)-1β and pituitary adenylate cyclase-activating polypeptide (PACAP)/vasoactive intestinal polypeptide (VIP) acting on the membrane receptor PAC-1R. In addition, BMAL1 acts on nuclear receptors (REV-ERB) which subsequently inhibits the invasiveness promoter circadian protein FABP7. Conversely, casein kinase1 (CK1) attains a tumor promoting activity through inhibition of PHLPP-1β.

Both the positive and negative limbs of the circadian molecular cycle are interlaced where BMAL1/CLOCK induces the expression of the nuclear receptor *REV-ERB*α which in turn acts as a transcriptional repressor of *BMAL1* creating a secondary TTFL cycle. Fatty acid binding protein 7, implicated in cell growth and differentiation, was reported to be a transcriptional target of REV-ERB and its transcription activating counterpart RORα suggesting an oscillatory expression profile. *In vitro*, proliferation and migration of GBM were enhanced upon suppressing REV-ERBα activity which led to increase the gene expression of fatty acid binding protein 7 (Figure 4). Similar results were obtained for proliferation aspects which was still compliant to *in vivo* studies. GBM were used in this study as a model to reflect neurogenesis related aspect however the endogenous presence of such circadian regulators in GBM may indicate a possible link to the control of various processes in these cells (91).

## The Clock and Glioma Pathways of Cell-Cycle Regulation, DNA-Damage Repairand Apoptosis

The genomic integrity is preserved through specialized systems which meticulously detect and resolve defects in the DNA ensuring low rates of spontaneous mutation during each cell generation, thus having tumor suppressor activity that needs to be perished for cancer to progress (92). Hence, during tumorigenesis DNA-maintenance machinery is bombarded by several mutational hits. Additionally, the apoptotic machinery which acting as a safe guard against accumulation of mutations is also compromised (92, 93). Since, both opposing processes are considered as the determinants of cell cycle progression, their disruption funnels into an uncontrollable proliferative capacity. Among the non-clock functions asserted to circadian genes is the regulation of cell cycle progression, genomic stability as well as DNA damage responses. Evidently, some of the genes controlling cell proliferation and cell cycle progression c-Myc, and *CYCLIN D1* are categorized amongst first- and second-order clock-controlled genes, hinting the relatedness of the two cycles (94, 95). Interestingly, recent reports have implicated the circadian controller Timeless protein in linking the cell cycle with the mammalian circadian rhythm in a model referred to as a “direct coupling” (96). Mechanistically, at G2/M checkpoint, human Timeless protein interacts with ataxia telangiectasia protein (ATR), a DNA damage sensor kinase, promoting the phosphorylation of Checkpoint kinase 1 leading to cell cycle arrest or apoptosis (97). Additionally, the down-regulation of the Timeless gene in human carcinoma cells shortened telomeres, revealing its importance in maintaining telomere length (96, 98). Relatedly, the protein kinase, WEE1 is cell cycle regulator which falls under the influence of the molecular circadian clock. This model is referred to as “serial coupling” under which *c-Myc*and*CYCLIN-D1* fall (97). Functionally, WEE1 is a serine/threonine as well as a tyrosine kinase which inhibits CDC2 (known as cyclin-dependent kinase 1) thus hindering G2/M (99). Such studies highlight the existence of coupling agents between cell cycle and cellular clock that are implicated in GBM pathogenesis.

Circadian genes may potentially influence glioma survival as the overexpression of *PER1 and PER2* was found to inhibit the growth and increases apoptosis in tumor cells (100, 101). Functionally, PER1 and PER2 sustain the normal cell cycle via regulating the expression of *P53 and c-Myc* (101) Supporting these data, other studies demonstrated that high levels of PER2 in cancer cell lines and glioma xenografts correlated with increased induction of P53 and apoptosis (102) and overexpression of *PER2* in irradiated glioma induces a decreased of c-Myc mRNA and protein levels (100). The *c-Myc* is repressed upon binding of P53 protein to its promoter (103). Furthermore, the down regulation of *PER2* expression promoted apoptosis in wild-type TP53 human glioma cells (U343) exposed to X-rays. The results were ascribed to the reduced expression of ataxia telangiectasia mutated (ATM) *and TP53*encoding genes, which regulate DNA damage and repair via the ATM-TP53 pathway. Increased expression of *c-Myc* was correlated to the apoptotic activity (104).

Melatonin is secreted by the pineal gland which acts as a gatekeeper of circadian rhythmicity. Circadian genes control melatonin production where the gene encoding N-acetyl transferase (the rate-limiting enzyme in the synthesis of melatonin) harbors an E-box element in its promoter, which is the site of BMAL1/ CLOCK heterodimer binding and transactivation. Several studies have showed the multi-faceted mechanisms underlying the potent anti-tumoral effect of melatonin against glioma, including estrogens inhibition, antioxidant stress reaction, and neuroprotective effect (105). Melatonin significantly inhibited *miR-155* expression in human glioma cell lines at concentrations (1 nM, 1, and 100 mM) with most inhibitory effect noticed at 1 mM. Treatment of U87 cells with 1 mM melatonin demonstrated significantly lower the capability migration and invasion compared with control cells. This is attributed to the inhibition of nuclear transcription factor, c-MYB. Melatonin oncostatic effect is mediated through apoptosis induction without affecting cell cycle distribution of U87 cells (106). This was corroborated by another study suggesting that melatonin sensitized human glioma cells to TRAIL-induced cell apoptosis. The effect occurs through the modulation of PKC activity which in turn decreases AKT activation. The repression of AKT tips of the balance toward apoptosis via increasing death receptor 5 levels and decreasing the antiapoptotic proteins survivin and BCL-2 (107). All the aforementioned studies highlight the crucial effects of melatonin as potent antitumorigenic agent even toward resistant glial subtypes.

**Figure 5:**
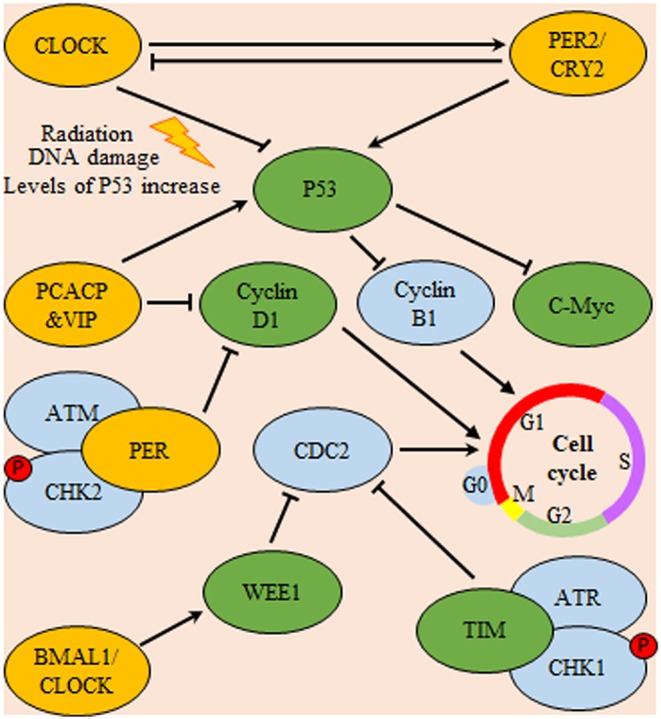
Circadian system regulation of cell-cycle progression in glioma. Expression of P53 is inhibited by Cl protein and promoted by PER/CRY heterodimers. In turn, P53 is a nodal regulator of both C-Myc and cyclin B1 (a regulator of cell-cycle progression through G1/S phase). Additionally, pituitary adenylate cyclase-activating polypeptide (PCACP) and vasoactive intestinal polypeptide (VIP) circadian regulating proteins control the cell-cycle progression via direct inhibition of cyclin D1 (a regulator of cell-cycle G0/G1 phase transition) and promotion of P53 mediated inhibition of cyclin B1. Also, both the circadian proteins TIM and Wee deter cell-cycle progression through G2/M check-point either directly or indirectly by inhibition of cyclin-dependent kinase 1 (CDC-2), respectively. Yellow color indicates main circadian regulators, green color indicates targets for circadian transcriptional factors and blue color indicates downstream effector molecules controlling cell-cycle progression.

As a controller of glioma cells proliferation, melatonin was found to control cell cycle progression through the up regulation of its cognate receptor (MT1) in PTEN wild type cells. This receptor up-regulation is mediated through the action of the circadian rhythm modulator early immediate gene c-FOS. PTEN is a PI3K phosphatase controlling the cell-cycle progression (108). In contrary to PER2 and melatonin antitumor effect, circadian genes might have antiapoptotic effect. Where, clock regulatory protein cryptochrome-2 (CRY2) was found to be associated with hampered radiosensitivity of C6 glioma cells. Compared with normal brain tissue, CRY2 mRNA and protein levels illustrated aberrant rhythmic periodicity of 8 h in glioma tissues. Upon irradiation, both mRNA and protein were increased at the zeitgeber time4 (nadir CRY2) and zeitgeber time8 (zenith CRY2) time points in gliomas. Immunohistochemistry experiments showed that high *CRY2* expression in glioma tissues was correlated with increased cell proliferation and decreased apoptosis (109). Similarly, *CLOCK* gene was repeatedly found to have tumor promoting action in glioma cells. Unlike control glioma cell line, apoptosis, and cell cycle arrest were increased upon silencing of *CLOCK* expression of in glioma cell line (U87). This action was attributed to alteration in the expression of apoptosis-associated genes. In comparison to control cells, levels P53 were increased, while those of c-Myc and Cyclin-B1 were decreased (110). This may explain the important role of Clock gene in inhibition of apoptosis through attenuation of pro-apoptotic signaling (110) (Figure 5).

Most of the biological processes are known to be interdisciplinary. This is best illustrated by the conjoint control of mitochondrial signaling over the intrinsic apoptotic pathway as well as cellular metabolic and respiratory pathways. Therefore, metabolism endure major reprogramming in tumorigenic environment such functional alterations are shall be highlighted here below.

## The Clock and Metabolism of Glioma

Glial metabolism was found to affect neuronal firing rhythms in SCN slices post applying fluorocitrate, glial metabolism inhibitor (2, 46). At the cellular level, circadian oscillators can be categorized into transcriptional clock and metabolic oscillators that temporally control a plethora of cellular processes. Intriguingly, redox/metabolic clock (namely peroxiredoxins oxidation cycle) is reported to independently function regardless of transcriptional oscillation (111). One recent study reported that, in T98G glioma cells the coordination between the canonical transcriptional and metabolic oscillators differs according to the proliferation status of cells under investigation. This was done through following the temporal variation of isotopically labeled glycerophospholipids metabolism as well as the cellular redox state. Overall results showed that both oscillators were found to be harmonious under *ex-vivo* arrest conditions however they got dissociated in proliferating cells (112). The circadian clock exercise its control over metabolism over distinct levels detailed below. Initially, mitochondrial gene expression as well as mitochondrial bioenergetic parameters that include membrane potential, cytochrome C oxidase activity exhibit diurnal oscillations (113, 114). Moreover, the mitochondrial level of NAD^+^/NADH oscillates, which in turn control mitochondrial protein acetylation via regulating the activity of mitochondrial sirtuins (SIRT) (115). Additionally, circadian clock regulates key metabolite levels namely ATP/ADP and cyclic AMP (114, 116). Physiologically, *in vivo*, circadian rhythms in ATP release appear to originate mainly from astrocytes within the SCN which is likely driven through calcium-dependent signaling (117). It was depicted that astrocytic extracellular ATP oscillations relay on key clock genes (*CLOCK, PER*, and BMAL1) as well as inositol triphosphate signaling suggestive of a clock controlled temporal increase in energy metabolism and glia activity (29).

Not only are the generation of ATP, but mitochondrial fission-fusion dynamics and mitochondrial bioenergetics clock controlled as well. Upon examining A127 human glioma cell-line post synchronization stimulus, the network morphology of mitochondrial showed circadian rhythmicity. Morphological rhythmicity was also matching the rhythmicity in content of ATP and the activity of OXPHOS. These circadian oscillations relied on rhythmic activity of dynamin-related protein 1 (DRP1), mediator of mitochondrial fission. Interestingly, retrograde signaling of DRP to the clock has been evident. Knocking out of *DRP1* gene resulted in suppression of circadian variation in the gene expression of clock activator *BMAL1* and repressors *PER1* and *PER2* (118). DRP1 was found to be a downstream molecule of key metabolic regulators including sirtuin (silent mating type information regulation 2 homolog) 1 (S. cerevisiae) (SIRT1), sirtuin (Silent Mating Type Information Regulation 2 Homolog) 3 (S. Cerevisiae) (SIRT3), and 5′ AMP-activated protein kinase (AMPK). This shows the intricate circadian control of metabolism via entwining crucial nutrient sensors like SIRT1, peroxisome proliferator-activated receptors (PPAR)-α, and AMPK with the clock machinery (119–121).

Since multiple connections between the circadian clock and cellular metabolism have been reported therefore, it can be concluded that cancer-related metabolic abnormalities may in part be due to disrupted circadian rhythms (122). This brings us to one more mechanism of circadian control of metabolism which occurs through controlling the expression of genes involved in metabolism (123). Experimental circadian disruption relevant to environmental cues like light-light cycle (LL) is considered for glioma tumor growth by promoting an anabolic metabolism processes where results indicated that key enzymes involved in lipogenesis (acetyl-coenzyme A carboxylase, fatty acid synthase, and PPAR-γ), and glucose uptake (Glucose transporter 1), were upregulated in the tumor microenvironment obtained from sLL *in-vivo* model. Findings also highlighted the concomitant increase in expression of *Glucose transporter 1 gene* together with the up-regulation of MYC favoring glucose metabolism, a main feature of tumorigenic metabolic reprogramming. LL tumors were highly infiltrated by macrophages as compared to light-dark cycle tumors. Tumor associated macrophages (TAMs) are conscripted by tumors to support and promote its growth. Thus, TAMs possibly might have contributed to the observed increased levels of the pro-angiogenic factor:vascular endothelial growth factor-α mRNA in LL glioma tumors (124).

Moreover, nicotinamide adenine dinucleotide (NAD) is fundamental coenzyme involved in cellular redox reactions as well as a cofactor for NAD-dependent enzymes. NAD-dependent enzymes, including sirtuins (SIRT1-7). Poly (ADP-ribose) polymerase (PARP), has an essential role in the maintenance of metabolic homeostasis DNA repair, and genomic stability. Furthermore, NAD acts as a co-regulator of circadian rhythms. NAD biosynthesis is regulated by a rate-limiting enzyme called nicotinamide phosphoribosyltransferase (NAMPT) in the salvage pathway. NAMPT proved to be a potential prognostic and therapeutic biomarker for GBM. Where, patients with higher expression of NAMPT exhibited worse prognosis (125). Another nodal co-regulator is peroxisome proliferator-activated receptor γ coactivator 1α (PGC1α) that integrate the clock of the body and energy metabolism (126). Recent studies showed that PGC1α was expressed in the GBM mitochondria. Consequently, genes involved in mitochondrial functions, including tricarboxylic acid cycle, lipogenesis, OXPHOS, and antioxidant genes, were up regulated in those cells (127).

Dysregulated PI3K/AKT signaling pathway might notably contribute to tumorigenesis. The activity of AKT is finely tuned by balance between activating kinases vs. deactivating phosphatases. Among the phosphatases, PH domain leucine-rich repeat protein phosphatase (PHLPP) splice variant *PHLPP-1*β also named suprachiasmatic nucleus circadian oscillatory protein mRNA levels oscillate in the SCN in a circadian fashion. PHLPP was found to terminate AKT signaling by direct dephosphorylation of the hydrophobic motif. Given its tumor suppressor effect, the PHLPP levels are markedly decreased in GBM cells that have elevated AKT phosphorylation. As shown by the results, albeit having comparable levels of AKT, LN444 GBM with reduced PHLPP relative to LN319 cell lines had dramatically increased phosphorylation levels of Ser473 and Thr308 (128). Among the circadian regulators, CK1 was found to phosphorylate PHLPP-1 generating a phosphodegron motif that directs PHLPP toward degradation (129).

**Figure 6:**
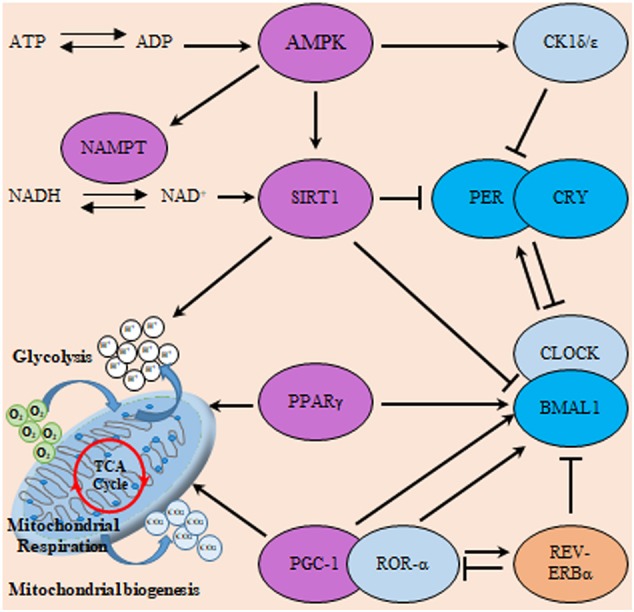
Interactions of the clock and metabolic regulators in glioma. The molecular clock TTFL operates through transcriptional activators (CLOCK, BMAL1, and RORα), transcriptional repressors (PER, CRY, and REV-ERB) as well as posttranslational regulators (CK1). This circadian clockwork orchestrates the expression of numerous metabolic regulatory genes to coordinate metabolism including sirtuin (silent mating type information regulation 2 homolog) 1 (S. cerevisiae) (SIRT1), ahistone deacetylase (HDAC) which counteracts CLOCK-mediated acetylation. Moreover, SIRT1 and CLOCK also modulate the acetylation status of PER and BMAL1. Additionally, energy-sensitive clock genes including BMAL1 and REV-ERBα receive direct regulatory feedback from transcription factors involved in metabolism including peroxisome proliferator-activated receptors (PPAR) and PGC-1α. In addition, cellular energy supply (as reflected in ATP/ADP and NAD+/NADH ratio) can directly influence clock activity via the induction of SIRT1 and 5′ AMP-activated protein kinase (AMPK) respectively. Where, AMPK modifies the clock directly through actions on CK1ε, SIRT1, and nicotinamide phosphoribosyltransferase (NAMPT). Violet color indicates energy sensitive regulatory molecules.

Another major player in the gliomagenesis is the WNT/β-catenin pathway which promotes the growth of GSCs, invasiveness, and resistance to therapy (130). The overexpression of *BMAL1* in glioma might in turn activate Wnt/β-catenin which can be also activated by PI3K/AKT pathway. Subsequent transcriptional activation of WNT/β-catenin downstream signaling cascade leads to the induction of aerobic glycolysis, which then promotes cell proliferation, angiogenesis, and anabolism of biomolecules in gliomas (131). Similarly, PPAR interferes with the mammalian clock and energy metabolism (132). PPAR-γ is a nuclear hormone receptor exhibiting transcriptional activity and is known to be implicated in glucose as well as lipid metabolism regulation (133). Reports indicate that, PPAR-γ activators induce cell cycle arrest and reduce local invasiveness in glioma. Conceivably, PPAR-γ is suppressed by the previously mentioned tumor promoting signaling PI3K/AKT (134). PPARs are rhythmically expressed in mammalian tissues (135), and directly interact with the genes of core clock. Deletion of PPAR-γ impairs diurnal rhythms (136). PPAR-γ agonists activate BMAL1 and the of CLOCK/BMAL1 heterodimer formation (137). Ultimately, the activation of PPAR-γ inhibits the WNT signaling. Explicably, PPAR-γ is reported to be down regulated in gliomas. A comprehensive scheme integrating metabolic regulators with the circadian TTFL machinery is illustrated in Figure 6.

Along the same vein correlating the cell-cycle/cell-differentiation, metabolism, and circadian clock, it was recently reported that apart from standard circadian circuitry, GSCs recruit circadian regulators for promotion of stemness maintenance and metabolism. Unlike differentiated GBM cells and non-malignant brain cultures that showed potent circadian rhythms, GSCs displayed intense dependence on core clock transcription factors, BMAL1, and CLOCK for optimal cell growth where induction of cell cycle arrest and apoptosis followed *BMAL1* or *CLOCK* downregulation (138), a modest anti-proliferative effect was observed upon knockdown of NPAS2, indicating a non-redundant function in GSCs. The circadian transcription factor BMAL1 was found to indispensable for stemness maintenance through the transcriptional activation of *SOX2, OLIG2*, and *MYC* stemness regulators. Moreover, unlike neural stem cells, BMAL1 got repurposed to control glucose metabolism and lipid biosynthesis via being recruited to H3K27ac epigenetically tagged metabolic genes in active chromatin regions of GSCs. The Targeting *CLOCK* or *BMAL1*, reduced the expression of the tricarboxylic acid cycle enzymes as well as attenuated mitochondrial metabolic function. Finally, the combinatorial use of CRY and REV-ERB agonists revealed a synergistic antitumor activity against GSCs (138). This study set the stage for considering the role of epigenetic modulations in gliomagenesis.

## The Clock and Epigenetic Modification in Glioma

Both the circadian clock and epigenetics act to integrate internal processes with environmental signals. Circadian clock affects rhythmic variation of epigenetic processes including methylation of genomic DNA, histone modifications, and non-coding RNA expression, (mainly miRNA). Concurrently, these epigenetic events can directly modulate the iterative transcription of core circadian genes and in-turn clock output genes (139). Thus, studying the interplay between circadian clock and epigenetics is crucial for better understanding of carcinogenesis. Since, promoter hypermethylation and histone deacetylation are prevalent epigenetic markers of tumor-associated transcriptional silencing. Conceivably, a myriad of tumor suppressor genes might fall under the influence of such epigenetic modifications in glioma. In a microarray analysis-based study investigating epigenetically silenced genes in malignant glioma trichostatin A (TSA), a pharmacologic histone deacetylase (HDAC) inhibitor, was utilized. TSA application hindered the silencing deacetylation process consequently promoting gene expression. Among others, BMAL1 gene was found to be sensitive to the pharmacological reversal of the epigenetic repression affirming its potential tumor suppressor effect. TSA treatment culminated into a remarkable arrest of cell growth, inhibition of cell cycle, activation of caspase, and cell death in human glioma T98 cells, U87 cells, and primary glioma cultures when relative to control-treated cells (140). Along the same vein, another study thought to investigate the possible epigenetic modification of PER2 gene in 92 human glioma specimens. Through employing immunohistochemical staining and methylation specific PCR the study revealed that *PER2* gene expression is deregulated in 52.17% of the glioma cells relative to the nearby non-cancerous cells which was attributed to promoter methylation (141).

Chromatin remodeling might stand as one of the links between an altered circadian clock and cellular metabolism. AKT acts as a nodal molecule interconnecting circadian control to epigenetic modulation. It was previously reported that elevated AKT activity lengthens circadian period. Also, it was found that, AKT activation is a crucial element of histone acetylation in cancers cells, which gives direct manifestation of epigenetic alternation for a specified metabolic enzyme. This investigation was confirmed using in AcH4 human glioma cells (142).

## The Chronotheraputic Drug Sensitivity in Glioma

Clocking the drug influences the clinical efficacy of cancer chemotherapeutics. Amongst the standard treatments of GBM is temozolomide (TMZ) DNA alkylating agent. Recent studies have shown that the cytotoxicity of TMZ could be modulated by cell-intrinsic circadian rhythms in GBM cells of both human and murine. The maximum TMZ efficacy in relation to apoptosis, DNA damage, and inhibition of cell growth was developed near the peak of *Bmal1* gene expression, as well as loss of *Bmal1* lessens the efficacy of TMZ (143). Results from another study carried out on rat glioma cell line (C6) and doxorubicin (anti-cancer agent) highlighted the significance of nuclear pore complex in cancer therapy and proposed that the mechanism of nuclear export and the maintenance genes of CSCs could be suppressed by inhibitors at appropriate phase while keeping the tumor-suppressing capacities of *PER2* expression (144).

Therapeutically, the adjuvant usage of curcumin with chemotherapeutic drugs including cisplatin or doxorubicin was found to induce apoptosis in GBM cells. From the molecular perspective, curcumin activates BMAL1 via stimulation of PPAR-γ. Studies propose that polyphenols (e.g., curcumin) activate SIRT1, an HDAC that controls circadian machinery. Circadian cell-death was attained upon the temporal administration of curcumin several hours prior to the rhythmic peak expression of mPER2 protein. These results revealed a temporal sensitivity which would be targeted in therapeutics based on curcumin or its analogs (145).

On a different aspect, 5-fluorouracil (5-FU) at 10 μM was found to disrupt the PER2 derived oscillation in C6 glioma cells. Furthermore, the amplitude if the rhythm was dampened as well. The therapeutic action of 5-FU might lead to the obtained observation due to the 5-FU inhibition of *de-novo* DNA and RNA synthesis (146).

## Circadian Clock As a Therapeutic Target for Gliomas

Extrapolating the spectrum of investigation, this section is dedicated to the drugging the clock thus affecting relevant clinical outcomes. Of relevance, C6 glioma cells were treated with clinically relevant valproic acid concentrations (0.5 or 1.0 mM) for a duration 1–7 days with the subsequent examination of expression levels of melatonin MT1 receptor along with selected epigenetic modulators. Valproic acid significantly affected the expression of MT1 receptor, HDACs1-3, and methyl CpG binding protein-2 in a time-dependent manner. Chromatin remodeling was further confirmed via TSA application which significantly induced *MT1 receptor* mRNA expression in a concentration-dependent pattern. The results imply that the oncostatic activity of valproic acid might be mediated through the upregulation of melatonin MT1 receptor (147). On another note, clock genes were assumed to be correlated with dopaminergic transmission, the main pharmacological target of antipsychotic drugs (APDs). Using U87 GBM cells, application of haloperidol (5 μM), first generation APD led to significant decrease in the expression of *CRY1* to almost 20-fold whereas, the *PER1* expression was increased by 1.5-fold. The observed findings may be produced via a signaling axis compromising D2-receptors and increasing CREB transcriptional factor phosphorylation (148).

A mini-screening study to investigate for drugs that can affect the period length using C6 glioma cells a model system. The results implicated several drugs to be significantly disrupt the measured parameter namely kinases inhibitors. Among the inhibitors, inhibitors against CK1 and CK2, the main mPER partners showed significant period length changing effect. Inhibitors against PI3K, JNK, AKT, and p38 MAPK were found to have a role as well. Such finding recaps the entire concepts demonstrated herein proving that the robustness of circadian rhythmicity is either controlled directly through the core clock protein making up the molecular TTFL or indirectly by other proteins implicated in various physiological proteins (149).

## Conclusion

The circadian machinery is a paramount regulator of intricate network of various signaling cascades influencing divergent biological processes. Intriguingly, circadian system plays milestone roles in brain tumors pathogenesis. Intervening with the circadian system deems attractive in the field of glioma research because it offers a versatile potential in impacting glioma on different fronts. Chronobiological application of therapeutics can effectively impinge onstem cells growth, invasiveness of tumor, angiogenesis, and drug delivery culminating into an improved therapeutic outcome.

## Future Prospects

At a clinical level, monitoring circadian parameters like body core temperature and sleep/wake cycles deems instrumental as both diagnostic and prognostic tool to assess gliomas. Circadian reprogramming using small-molecule drugs to modulate circadian proteins in GSCs might offer a selective treatment approach for aggressive gliomas. This can hinder circadian-related changes in gene expression profiles selectively in cancer cells without perturbing the clock in normal ones. Glioblastoma seems to be a good candidate for chrono chemotherapy through the integration of circadian biology data to develop effective treatment protocols. Accordingly, optimizing the regimen of drug administration will lead to improving the efficacy and reducing the toxicity of chemotherapy. Employing circadian clock modulating drugs as adjuvant therapy along with standard chemotherapeutic agents might be impactful in reducing their doses to safer levels. Thorough research is still needed to study the potential off-target effects of using clock modulating drugs as circadian clock has wide systemic effects.

## Author Contributions

KA and ME formulated the topic of the review and contributed to the final draft of the review. KA wrote the first draft of the review.

### Conflict of Interest

The authors declare that the research was conducted in the absence of any commercial or financial relationships that could be construed as a potential conflict of interest.
